# Advising caution in studying seasonal oscillations in crime rates

**DOI:** 10.1371/journal.pone.0185432

**Published:** 2017-09-22

**Authors:** Kun Dong, Yunbai Cao, Beatrice Siercke, Matthew Wilber, Scott G. McCalla

**Affiliations:** 1 Center for Applied Math, Cornell University, Ithaca, NY, United States of America; 2 Department of Mathematics, University of Wisconsin–Madison, Madison, WI, United States of America; 3 Department of Mathematics, University of California, Los Angeles, Los Angeles, CA, United States of America; 4 Department of Mathematics, Harvey Mudd College, Claremont, CA, United States of America; 5 Department of Mathematical Sciences, Montana State University, Bozeman, MT, United States of America; University of Texas at San Antonio, UNITED STATES

## Abstract

Most types of crime are known to exhibit seasonal oscillations, yet the annual variations in the amplitude of this seasonality and their causes are still uncertain. Using a large collection of data from the Houston and Los Angeles Metropolitan areas, we extract and study the seasonal variations in aggravated assault, break in and theft from vehicles, burglary, grand theft auto, rape, robbery, theft, and vandalism for many years from the raw daily data. Our approach allows us to see various long term and seasonal trends and aberrations in crime rates that have not been reported before. We then apply an ecologically motivated stochastic differential equation to reproduce the data. Our model relies only on social interaction terms, and not on any exigent factors, to reproduce both the seasonality, and the seasonal aberrations observed in our data set. Furthermore, the stochasticity in the system is sufficient to reproduce the variations seen in the seasonal oscillations from year to year. Researchers should be very careful about trying to correlate these oscillations with external factors.

## Introduction

Rising temperatures are inspiring various cities and nations to examine the expected results of global warming on social behavior, and generally the ability of mankind to thrive on a hotter planet. As an example Thriving in a Hotter LA, a UCLA sponsored program in response to the President’s request for solving Grand Challenges, strives to pro-actively stem the negative effects of climate change through social change in Los Angeles. While there is evidence that large aberrations in temperature can lead to a measurable change in human hostility [1], the correlation between crime rates and seasonal temperature variations, believed to be important in the criminology literature [2], is poorly understood. Considering one of the most noticeable consequences of global warming is a shift in weather patterns, either locally warming or cooling, there is a need to understand how crime rates may change in response to the new weather patterns.

By applying a nonparametric analysis to daily crime data collected from Los Angeles, controlled by the LAPD and available in the S1 File, and Houston [3], we are able to extract and decouple the seasonal oscillations in crime rates from the long term trends and higher order oscillations in the number of daily crimes committed. As a result, we are able to extract variations in the amplitude of the seasonal oscillation from year to year. We strongly caution against seeking correlations between these annual variations in the seasonal amplitude and exogeneous factors, such as temperature: the underlying noise and randomness in this data is likely the leading order component in this variation. By seeking external correlations, a researcher is at high risk for finding a spurious correlation and p-hacking. To illustrate this phenomenon, we employ a simple stochastic model that reproduces the variations in crime’s seasonal rates. The main point is that any stochastic model that allows for the requisite periodic oscillations is likely to exhibit annual variations in the amplitude of the seasonal crime oscillation. Crime rates are strongly dependent on stochastic, and societal factors and this can easily overwhelm any correlation with an external forcing variable. The stochastic fluctuations are large compared to the dynamics and obfuscate the correlations between seasonal temperature fluctuations and seasonal crime rates. In some sense, the temperature dependence of crime’s seasonal behavior, unlike those for large scale human conflict, are a higher order effect when compared to stochastic and sociological factors.

Crime rates change with the seasons. This well-known fact has been the subject of criminology research for over a century [4, 5]. For example, every January burglary rates in Houston will be at a low, then during the summer they will peak. Though this is well known, the mechanism that produces this seasonality, the time of year that crime peaks, and whether all crimes display these oscillations is still debated [6–9]. These oscillations have implications in how police officers should be deployed, and how the success or failure of a police force in a particular district is measured [10]. Unfortunately the exact form of the oscillations is obscured by the nature of the data. First, crime in the United States has been in steady decline for the last few decades, and is at an exceptionally low level for most types of crime. Thus there is a very high level of noise compared to the average crime rate. This is one likely cause for the controversy in the seasonality of crime. For example, several studies have concluded rape is seasonal [2, 10, 11], while other studies find no strong evidence for a seasonal component [12]. Rape, however, is rarely reported and the number of daily cases is minimal [13–16]. The decrease in crime can additionally obscure a seasonal peak or shift its location in time [17]. Our first goal is to separate the seasonal component from the long term trends in crime directly from the noisy data. Once the nonlinear trend is removed, we can see the variations in the seasonal amplitude from year to year. Once we have a seasonal component, we then apply an ecologically motivated stochastic differential equation (SDE) based on the Lotka–Volterra model for predator-prey relationships to reproduce the seasonal variations. From this, we draw the conclusion that stochastic noise of the order seen in the crime data overwhelms the dynamics in our simple SDE model and creates the same sort of annual variations that appear in the data. We then caution against trying to seek high order correlations between weather trends and the changes in the amplitude of these seasonal variations from year to year. The stochastic terms are strong enough such that they can easily change the underlying dynamics and overwhelm any actual weather or temperature dependence.

Considering the complexity of human behavior and the noisy crime data, correlating annual rises in crime with a single exogenous variable, such as temperature, is an optimistic oversimplification. Crime data has enough noise and variations that by looking at the right data set any desired correlation can be found. However, there is ample evidence that unusually large jumps in temperature, such as an unseasonably hot day, do lead to rises in human aggression [1]: this is related to temperatures above the normal average during a given era, rather than seasonal high temperatures that are gradual, expected, and adapted to by people every year. While we believe variations in crime rates will be affected by the weather, our goal is to show that variations in the amplitude of the seasonal oscillation in crime from year to year could arise solely from endogenous factors, stochasticity in the system, and large social catastrophes. The variations from the randomness are larger than those that would be induced by temperature alone. One clear example in the data is the dramatic rise in property crime rates and burglaries in the end of 2008 after the financial crisis. External periodic forcing, however, is often a critical driver for seasonal oscillations, and many examples of this can be found in the literature [18, 19]. Our interest is in human behavioral response to these slow seasonal variations. For example, if unusually high average temperatures in one particular summer lead to unusually high aggravated assault rates in that summer and fall. We address this question in detail through a study of Los Angeles crime rates over a decade, as well as Houston crime rates over a three year period.

We compare the crime data to a minimalistic and ecologically motivated system of SDEs. Our goal is to show that a small amount of noise in these SDEs can produce the same annual variations in the amplitude of the seasonal component as is seen in the data. The SDEs serve as a model for human criminal behavior. The SDE system is based on the Lotka–Volterra equations [20, 21], a standard predator-prey system that exhibits periodic behavior with the fewest possible species and that has been used in the criminology literature [22]. The random variations in our model are sufficient to explain the annual variations in the amplitude of the seasonal crime oscillation. While our model is simplistic, it helps illustrate that even small stochastic variations for an underlying system with periodic orbits can exhibit the same variability that is seen in the data even without external forcing. Specifically, no terms specifically related to changing weather patterns are needed to produce the aberrations in the data. This implies that seeking correlations with external factors may produce spurious results. Because of the low crime rate, temperature variations certainly are not the leading order effect in the data. The stochasticity of human behavior overwhelms the small effects from temperature fluctuations. Though an increase in violence and civil unrest is likely to occur once global temperatures adequately shift [1], the seasonal variations from changing weather patterns are unlikely to dominate yearly crime rates.

## Extracting crime trends and seasonality

The raw crime data is exceedingly noisy. This noise completely obscures our ability to readily identify seasonal oscillations. Compounding the problem further, crime trends are falling in an inconsistent manner; over a single season, average rates can fall on the same order as the amplitude of the seasonal oscillations. There has been a long-term sustained reduction in crime [23, 24] in the United States. This downward trend is obscured by the annual, or seasonal, oscillations in crime rates, the variations in crime by day of the week, and the stochasticity in crimes that occur from day to day. The total number of crimes on any given day is quite low. For example the total number of property crimes in Los Angeles over an eight year period never exceeds 350 events on a single day, and can vary by 100 events from day to day. Houston generally records less than ten rape cases a day. Thus there is a great deal of noise compared to the signal strength.

Extracting a seasonal component and long-term trend from such a signal is therefore non-trivial. Most studies rely on crime data binned into monthly totals over many years [8]. They then conclude that seasonality exists from consistent differences in the highest crime month to the lowest crime month across the entire data set. Our study necessitates directly extracting the seasonal variations and the long term general trends from the daily crime data. This enables a comparison between crime seasonality in Houston and Los Angeles, as well as a detailed study of the annual variations in the seasonal oscillations that is otherwise unfeasible from the monthly binned data.

**Fig 1:**
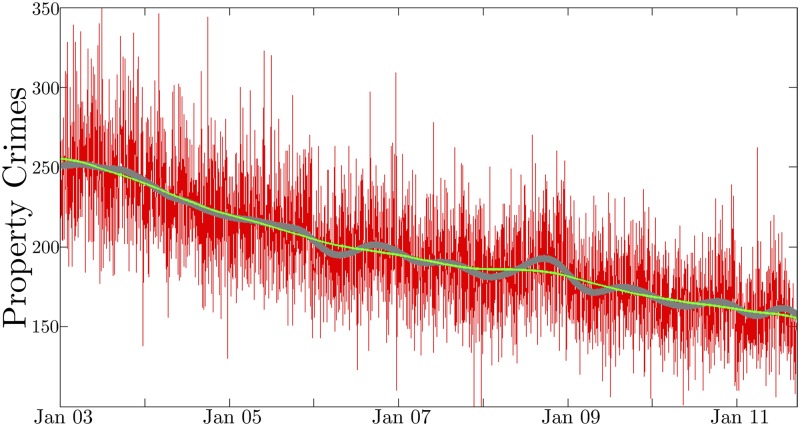
Property crime data. The daily aggregate property crime for Los Angeles is plotted in red. The gray curve is the SSA attained trend plus the seasonal term, and the green curve on top is the long term trend. Despite the relatively large number of daily crimes, the data is very noisy, and the seasonal variations are difficult to detect without applying some signal processing techniques. Throughout the rest of the paper, the plotted data will be smoothed with a moving window average after removing the trend to clarify and emphasize the seasonal oscillations in the later figures. Note that the SSA decomposition is always performed on the unsmoothed noisy data.

**Fig 2:**
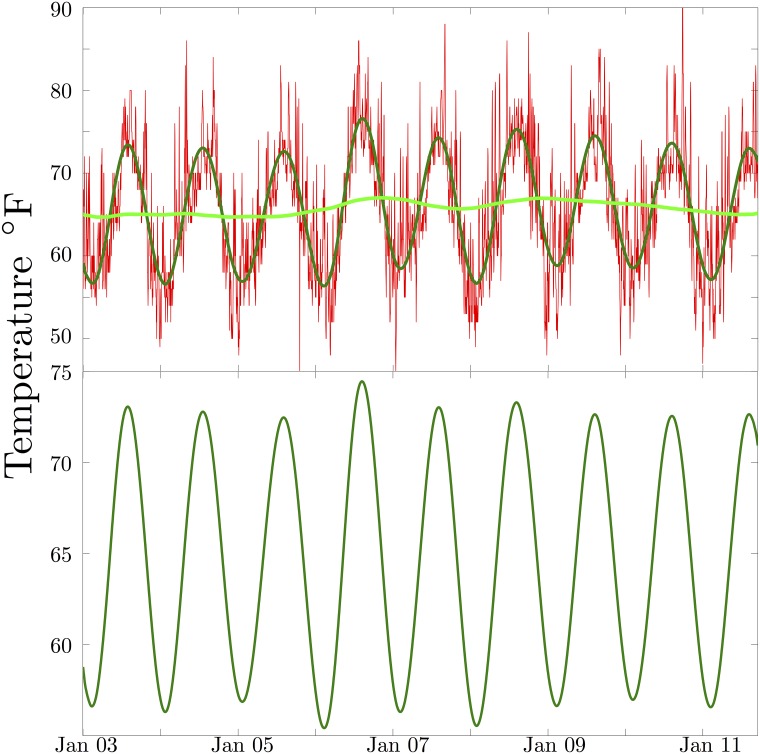
Los Angeles temperature data. In the top panel, the daily average of high and low temperature for Los Angeles is plotted in red. The green curve is the SSA trend, and the dark green curve is the seasonal component plus the trend. In the bottom panel, the seasonal component alone is shown. The temperature variations in Los Angeles are extremely regular, though the summer of 2006 does appear unusually warm. The aberrations in crime seasonality are far larger, and less consistent than the temperature variations.

Singular spectrum analysis (SSA), a nonparametric singular value decomposition (SVD) based technique, is used in meteorology to extract the long-term trends from noisy weather data. It also provides a natural decomposition of temporal data into components that vary on similar time scales [25]. SSA naturally separates time scales and allows us to differentiate between long term trends and the shorter term oscillations and noise in the crime data (Fig 1) and the weather data (Fig 2). These techniques can also be used to forecast future states of noisy data sets [26]. We apply SSA to daily crime data for Houston and Los Angeles. The data consists of the number of crimes that occur each day over several years. It should be mentioned that crime is known to vary by day of the week, and certain crimes are more likely on weekends or weekdays. Though SSA will extract these changes, we consider them lower order oscillations for the current study as they vary on a far shorter time scale than we are interested in.

The first step in the SSA involves creating a trajectory matrix. For a time series *X* = (*x*_1_, *x*_2_, …, *x*_*N*_) and a window length *L* = 365, we define the trajectory matrix as

$$\begin{array}{c} X=(\begin{array}{ccccc} x_{1} & x_{2} & x_{3} & \cdots & x_{K}\\ x_{2} & x_{3} & x_{4} & \cdots & x_{K+1}\\ x_{3} & x_{4} & x_{5} & \cdots & x_{K+2}\\ \vdots & \vdots & \vdots & \ddots & \vdots \\ x_{L} & x_{L+1} & x_{L+2} & \cdots & x_{N} \end{array}) \end{array}$$ (1)

with *K* = *N* − *L* + 1. Then a singular value decomposition (SVD) is performed on the trajectory matrix, providing a decomposition *X* = *X*_1_ + ⋯ + *X*_*L*_. Each of our modes then comes from averaging these matrices *X*_*i*_ along their anti-diagonal. The mode with the largest singular value always corresponds to the average crime trend. We then typically would look for any modes with an annual periodicity in the first twenty singular values and sum these to produce our seasonal component.

**Fig 3:**
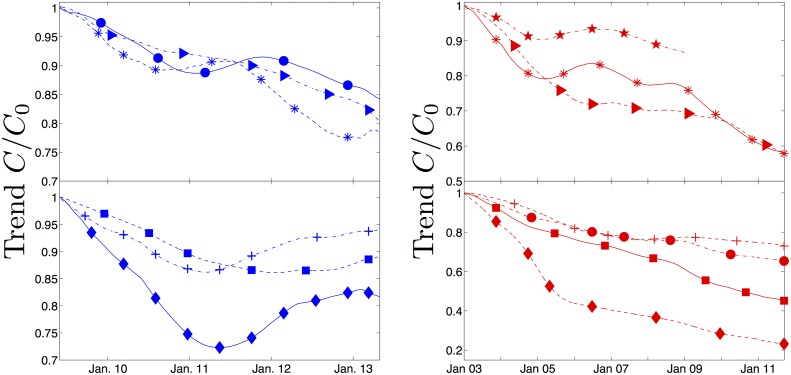
Crime trends. The long term crime trends *C*(*t*), or first mode in the SSA decomposition, for Houston (blue on left) and Los Angeles (red on right). The data is normalized to be the fraction of the initial crime level for the first time point *C*_0_. Top panel on left is aggravated assault (triangle), rape (asterisk), and burglary (circle). Top panel on right is break in and theft from vehicles (triangle), robbery (asterisk), and vandalism (star). On the bottom left is auto theft (+), theft (square), robbery (diamond). On bottom right is aggravated assault (diamond), burglary (circle), grand theft auto (square), and theft (+).

**Fig 4:**
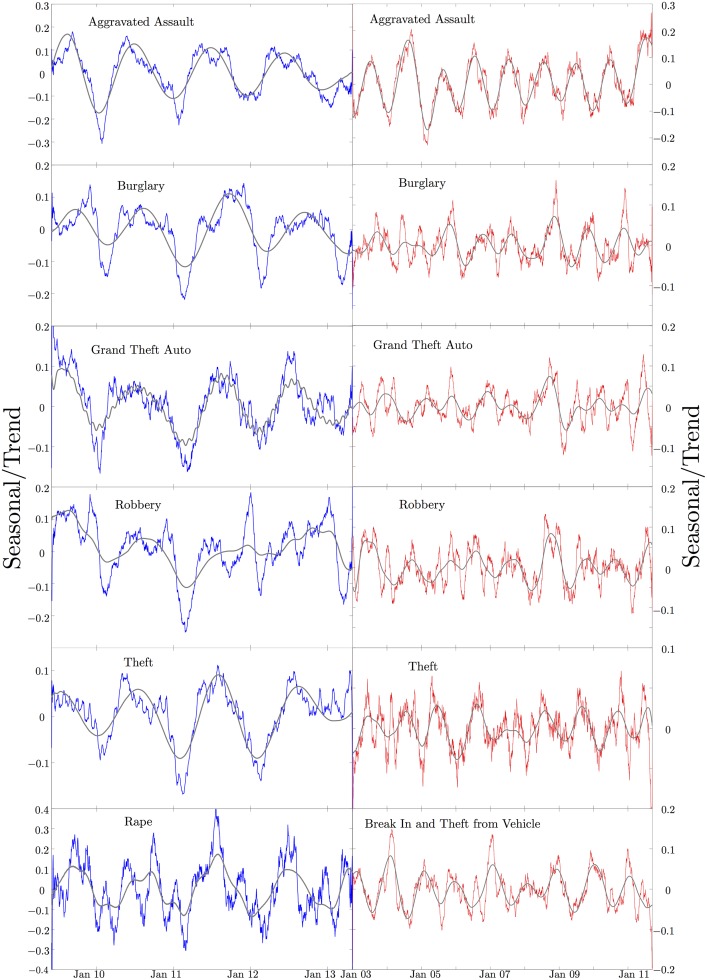
Seasonal crime component. A comparison between the raw crime data and the SSA seasonal component (grey) for Houston (blue) and Los Angeles (red). The trend is subtracted from the data and then the data is smoothed using a moving window average. As the oscillations are related to the total amount of crime, the data and seasonal component have been divided by the trend pointwise in time to normalize the oscillations. Note that the seasonal oscillations are on the same order compared to the trend level of crime across all crime types. Even when the level of crime is low, a fairly consistent oscillation is seen. Note the large aberration in the Los Angeles data just after the 2008 crash. Such social catastrophes can override any seasonality and lead to dramatic changes in future crime rates.

We emphasize that the most important term in our SSA decomposition is the trend, or first mode (Fig 3). Once this trend has been extracted, the data clearly exhibits seasonal oscillations once it has been smoothed with a moving window average. These same oscillations are not as clear without extracting the trend first. As is seen in Fig 4, our seasonal component provides a reasonable smoothed approximation of the data but in many cases loses some of the structure of the annual oscillations.

In Fig 3 the long term average crime trend from the SSA decomposition is plotted for Houston and Los Angeles. We see a general decrease in all types of crime in both cities, but there are several salient features of the data that are worth mentioning. First, crime decreases are not consistent in any sense. For every data set examined, the final level is lower than the initial level. However, this does not occur in a homogeneous fashion. Many crimes more recently seem on the rise in Houston. Second, the rate of decrease in Los Angeles seems to be slowing. After January 2006, the trend reduction appears comparatively small for all observed crimes. One possible cause is that crime is at a very low basal level, and it is hard to decrease it below this level. If this is the case, public funds applied towards lowering crime further will likely suffer from diminishing returns. Alternatively, police forces have not generally increased in size with respect to the population so it is likely difficult for the police to reduce the crime any lower without increased resources.

**Fig 5:**
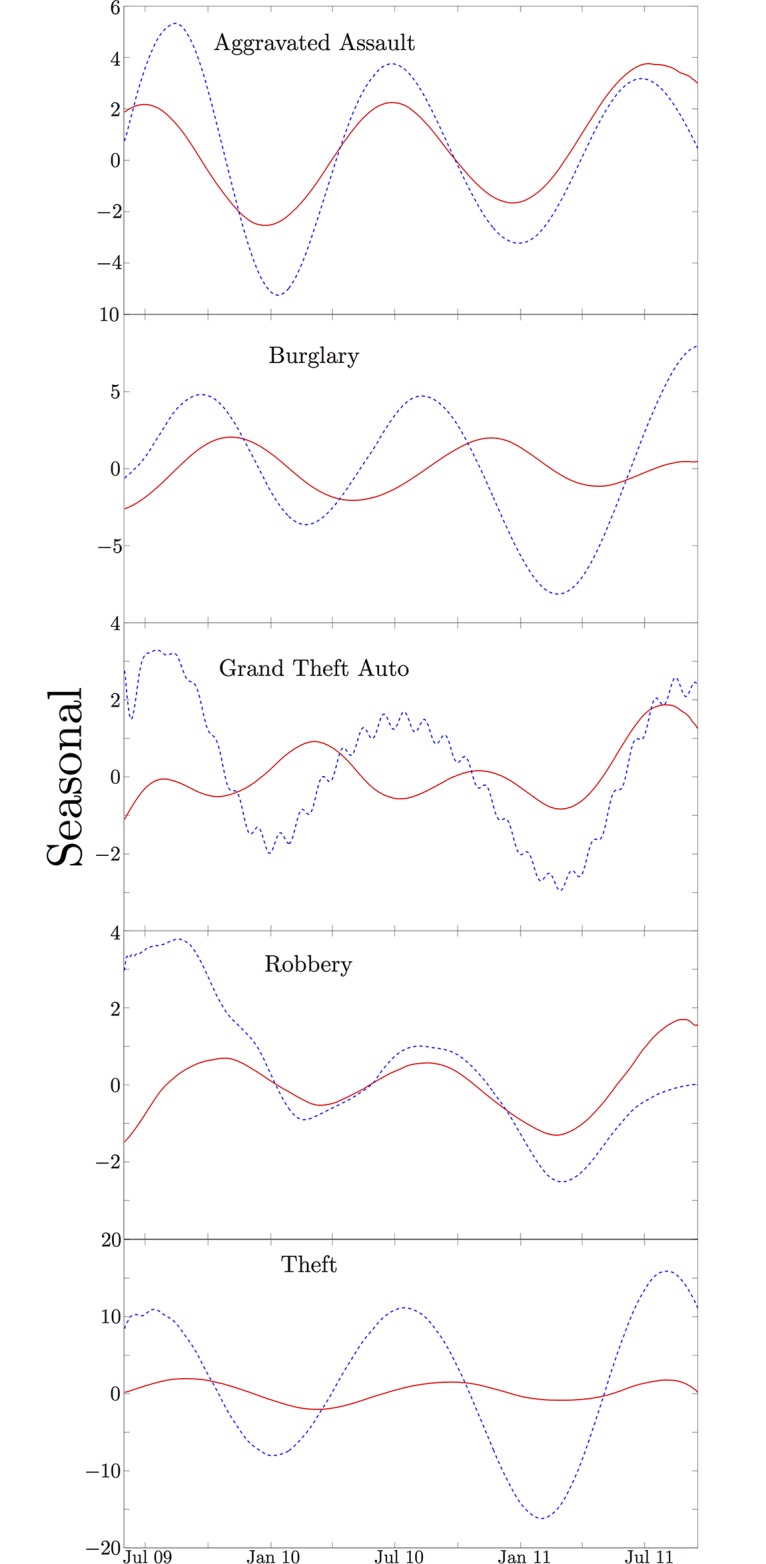
Differences in seasonality. Comparison between the SSA derived seasonal component from Los Angeles (red) and Houston (blue).

In Fig 4, the seasonal components are compared to the data. Note that for the SSA derived seasonal components, we are generally keeping only the leading order modes in our reconstruction of the seasonal data. This dramatically smooths the data, but loses some of the higher order effects in the seasonal components. However once the data is flattened by removing the trend, the raw data smoothed with a moving window average clearly shows seasonal oscillations for comparison. We will eventually compare both this smoothed average from the raw data and the SSA derived seasonal components to the proposed SDE model. We see a seasonal oscillation across all of these data sets, though this can be a little deceptive. Rape in Houston appears to have a seasonal component, but this occurs at an incredibly low amplitude and is nearly impossible to see in the raw data. Fig 5 compares the extracted seasonal components from Los Angeles and Houston. It is clear from the figures that in some cases, such as aggravated assault and robbery, the seasonal amplitudes between the two cities are synchronized. Grand theft auto, burglary, and theft appear to have a different seasonality between the two cities. Grand theft auto in Los Angeles actually appears to have an oscillation on the order of half a year rather than annually. While Houston seems to have a fairly consistent peak around July for most crime types, the seasonal peaks for different crimes in Los Angeles vary significantly.

## An ecological model for seasonal crime rates

Criminal encounters have an analogous relationship to predator-prey interactions. Exploiting this analogy has led to great success in recent years. In fact, Ref. [27] has recommended that mathematicians exploit this analogy further by concentrating specifically on modeling crime occurrences rather than criminal behavior. The authors in [28] adapt a model for coyote territories from [29] to understand gang territories. Similarly, employing agent based models to criminals has led to a better understanding of gang rivalries [30] and hot spot formation for burglary [31–34]. Ecological models have also successfully deepened our understanding of gang activity and territorial boundaries [22]. In these studies, criminals are generally treated with the same mathematical formalism as predators.

The best known of these ecological models, the Lotka–Volterra predator-prey equations [20, 21], exhibits periodically oscillating populations corresponding to a nonlinear center. In a nonlinear center, every possible initial condition leads to a periodic orbit, or in other words, to an oscillation similar to the seasonal oscillations in our crime data. The Lotka–Volterra equations are a two-component system of nonlinear, first-order equations. For a system of ordinary differential equations without external forcing, a two-component first order system is the simplest system that can exhibit periodic behavior and higher dimensional systems that exhibit periodic behavior can often be reduced to two-component systems on their center manifold. It is also important to note that external forcing can always be replaced by adding variables to the nonlinear system, so even these systems at best reduce to a two-component, first-order system.

We use the Lotka-Volterra equations as the basis for our crime model because of their past appearances in the criminology literature and further note that they are a minimal ecological model that exhibits the requisite periodicity. Furthermore, systems that exhibit families of periodic orbits, and do not exhibit a drifting trend, will robustly reduce to a Lotka–Volterra type equation when expanded to quadratic order. We reiterate that our modeling goal is to demonstrate that moderate stochasticity in a system that possesses the periodicity seen in the crime data is likely to exhibit large annual variations in the seasonal amplitude. Any more complex model will exhibit the same behavior if the randomness is comparable in size to the leading order deterministic dynamics.

The analogy between this ecological model and crime is as follows. In a given time period, the predator population is the number of crimes that occur and the prey is the number of possible targets available. In any criminal event there is a victim and offender, and their interaction probability scales, according to standard mass action kinetics, like the product of the two populations. The multiplicative constants in our model *β* and *δ* account for the likelihood of losing possible victims after a criminal encounter and a crime being committed during the encounter, respectively. In the absence of targets, we assume the crime rate will decay to zero at rate −*γ*. Similarly, the number of available victims will grow in the absence of crime with rate *α*. In principle the number of available targets for victims is very large. However only a small fraction of them will occur in a suitable setting and display suitable characteristics to be victimized. For assault, rape, or robbery, the assailant only will encounter a limited number of suitable victims in a vulnerable location. Burglars are known to preferentially search a limited set of possible homes, generally within a block of previous successful burglary sites. In our model, the number of available targets, or prey animals, *x* is then generally kept small (on the order of 15). We note that this number must be estimated from sociological data, and cannot be directly measured from the daily crime data. The number of crimes that occur is then the predator variable *y*. In our analogy, victims disappear from the system after a crime occurs. When an area has recently hosted a number of crimes, people are far less likely to go out thus the number of possible victims will be decreased. Our model adds multiplicative noise, such as used in the derivation of the Black–Scholes equation for European Call options, onto the standard Lotka–Volterra equations:

$$\begin{array}{cc} dX_{t}=X_{t}\left(\alpha -\beta Y_{t}\right)\;dt+\sigma_{1}X_{t}\;dW_{t}, & \;X(0)=X_{0}\\ dY_{t}=-Y_{t}\left(\gamma -\delta X_{t}\right)\;dt+\sigma_{2}Y_{t}\;dV_{t}, & \;Y(0)=Y_{0}. \end{array}$$ (2)

We note that this is in Itô form, with the standard interpretation that the system only has information about the past and not the future. This allows for modeling the random variations in the number of targets and crimes from day to day. The noise from the geometric Brownian motion is proportional to the total number of crimes and targets, and helps ensure positivity of the predator and prey populations. Positivity follows from the invariance of the *X* and *Y* axes under the SDE evolution, and continuity of sample paths. The dynamics of the deterministic Lotka-Volterra equations can be reduced to a one parameter family after non-dimensionalization. Using the change of variables $\tilde{X}=\delta X/\gamma$, $\tilde{Y}=\beta Y/\alpha$, and *τ* = *αt* we arrive at

$$\begin{array}{c} \frac{d\tilde{X}}{d\tau }=\tilde{X}\left(1-\tilde{Y}\right)\\ \frac{d\tilde{Y}}{d\tau }=-\tilde{\gamma }\tilde{Y}\left(1-\tilde{X}\right) \end{array}$$

where our single parameter is $\tilde{\gamma }=\gamma /\alpha$.

In order to estimate the deterministic parameters, we first use least squares to fit the deterministic Lotka–Volterra equation to the seasonal component of the crime data as extracted from the SSA analysis. Our initial parameters for this minimization can be estimated as follows. The average crime rate $\bar{Y}$ determines the relation $\bar{Y}=\alpha /\beta$, and our estimate on average number of targets $\bar{X}$ (which we believe to be small and on the order of 15) determines $\bar{X}=\gamma /\delta$. We further reduce our parameters by assuming *β* = *δ*, or that a single crime will remove a single victim. Our periodicity should be on the order of a year, and we can estimate the time period by appealing to the linearization around the fixed point to find $T=365\approx 2\pi /\sqrt{\alpha \gamma }$. For the stochastic component, we fix *σ*_1_ = 0 and only allow for stochastic jumps in the crime variable; these stochastic parameters could in principle be extracted using a maximum likelihood estimator. For the initial data, we assume the oscillations are approximately elliptical. This allows us to estimate where in the phase space of the deterministic system our initial data lies with respect to the oscillations in the crime *Y* variable.

**Fig 6:**
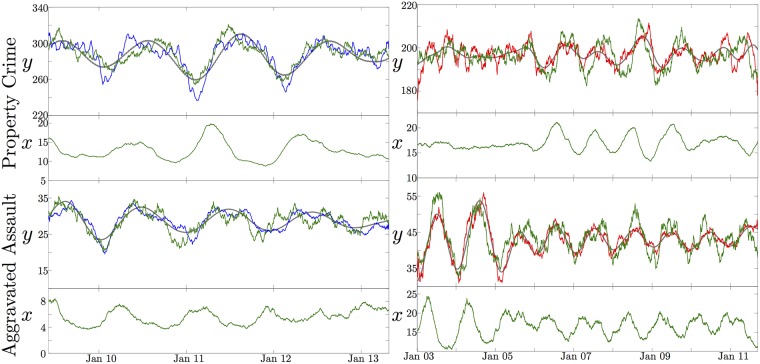
Stochastic model comparison. A comparison between eq (2) (green) and the moving window averaged aggravated assault and property crime data for Houston (blue on left) and Los Angeles (red on right). The stochastic differential equation is able to reproduce the seasonal component across various crime types and locations, as well as its annual variations without relying on any exogenous factors. Aggravated assault simulation parameters: Houston *α* = 28.78, *β* = 17.20 = *δ*, *γ* = 0.0799, *σ*_1_ = 0, *σ*_2_ = 0.07; Los Angeles *α* = 42.81, *β* = 8.29 = *δ*, *γ* = 0.09, *σ*_1_ = 0, *σ*_2_ = 0.05. Property crime parameters: Houston *α* = 286.82, *β* = 23.79 = *δ*, *γ* = 0.02, *σ*_1_ = 0, *σ*_2_ = 0.07; Los Angeles *α* = 197.14, *β* = 4.88 = *δ*, *γ* = 0.06, *σ*_1_ = 0, *σ*_2_ = 0.03.

In Fig 6, a comparison between eq (2) and the smoothed experimental data can be seen for property crimes and aggravated assault in both Los Angeles and Houston. We see that the data can be reasonably reproduced with our stochastic model, including the large shift in annual amplitude for the seasonal component. Additionally, the target population remains relatively small, on the order of 10–30, rather than the hundreds or thousands, as was in line with our above interpretation. A typical run will produce noisy, periodic oscillations with varying amplitudes but will not reproduce the data nearly as well as the examples in Fig 6. Individual runs of a stochastic system will generally exhibit a large degree of variability, but this does imply that our model can exhibit the variations seen in the data. These shifts in amplitude, as well as the missed periods seen in the Los Angeles data around 2004 to 2006 and in 2008 and 2010, are quite typical in our model. This implies that stochasticity is sufficient to produce the variations in crime seasonality; exogenous factors are not necessarily the cause of this variability in amplitude from one year to the next. External forcing should be an important factor in the seasonal trends, however it is not the only source of the variations in these crime rates. The social ecological terms alone are sufficient to explain the seasonal shifts in crime rates, and searching for correlations between varying seasonal crime rates and various weather patterns is likely to produce incorrect conclusions. Crime is simply too stochastic, and the data too noisy, for such correlations to be useful. We do want to caution against using (2) to make forward predictions for future crime rates. We believe a better approach is to use the SSA decomposition to predict the future crime rates.

The Los Angeles property crime data, as seen in Fig 6, illustrates another important feature for these systems. Frequently, an entire seasonal oscillation seems missing. This can easily be explained in terms of stochastic models with underlying periodic orbits. The stochastic terms can effectively overwhelm the deterministic evolution enough to push the system to very small periodic orbits near an equilibrium state. The periodicity is then masked by the random noise around the fixed point. Eventually, the random stochastic kicks will push the system back into a larger orbit and the deterministic terms will dominate. In our model, this is exhibited as periods of seasonal oscillations mixed with periods of near constant behavior. Additionally, the variations in the amplitude of the seasonal cycle from year to year arise from this mechanism, rather than from any temperature based forcing. For a single limit cycle, [35] found that moderate noise, comparable to the strength of the limit cycle, can force the system to propagate opposite to the limit cycle rotation and can also stabilize an otherwise unstable equilibrium. For an entire family of periodic orbits, when the system is near the equilibrium any source of noise will dominate the deterministic system and drive the orbit towards larger oscillations. Eventually the deterministic oscillation and the stochastic noise will be comparable, and effects similar to those for the limit cycle from [35] will be observable. This balance between the periodic orbit and the stochastic forcing can persist for long times. Eventually the stochastic terms in the Lotka–Volterra model will become higher order and the system will slowly evolve towards a state exhibiting larger and larger oscillations. In the biology literature, there is extensive study on noise stabilized oscillations: these typically involve stable spiral fixed points where some form of stochasticity creates periodic oscillations [36, 37]. The stochasticity is the only reason the system does not appear stationary in time. This is effectively the same mechanism that produces the annual variations in the crime seasonal swings.

## Discussion

An underlying belief in the criminology community is that the seasonal oscillations are often driven by an external force, such as temperature or rainfall. The idea is that people are more aggressive during warmer seasons, and certainly that weather patterns like heavy rainfall can alter human behavior. Los Angeles, however, is extremely temperate with a short season for rainfall. The seasonal variations in weather are extremely consistent over the time period we study; see Fig 2. Los Angeles still displays seasonal variations in crime rates with a significant amount of variation in the year to year swings. Additionally, different crimes peak at different times of the year, and the same crimes peak at different times in LA and Houston. Another salient feature of the data is pronounced shifts in the amplitude of the seasonal oscillation from year to year. The amplitude can shift significantly between two consecutive years, and this has been used as a basis to seek external factors that correlate well with these shifts. If an underlying factor such as temperature is responsible, then these variations can be explained by looking for correlations with these external factors. We see that this variation can be explained by a small amount of stochastic noise shifting the periodic cycles in the system and need not be derived from an external control parameter. Because such stochasticity can create dramatic variations in annual crime oscillations, we caution criminologists against seeking correlations between this data and exogenous factors.

One of the major difficulties in extracting the seasonality of crime for the United States is the low number of daily crimes and high variability in crime from day to day. As can be seen in Fig 3, the drop in crime has not been monotone. Various lulls in crime reduction have been seen, such as from 2005 to 2009 in Los Angeles, and the rate of reduction is slowing. Some crimes have actually increased in prevalence during part of the examined time intervals. A standard technique to evaluate the effectiveness of police departments is to look at the changes in various crime rates over a quarter. Given crime rates are already so low and known to oscillate on such a time scale, this is not an ideal measure of success for the police. However using the SSA decomposition, the expected daily crime trends can be forecast from previous data and used as a benchmark against which to examine the actual crime rates. However, compounding the low level of crime and non-monotone trends with the seasonal shifts, it really is necessary to examine crime levels over several years to understand whether any policing strategy is effective. We see that the annual seasonal variations change significantly each year, and the overall trends change on a small enough scale that they can be overwhelmed by the oscillations and noise in the system.

When various different crimes peak in different seasons, the police can concentrate resources on limiting the crime that is most prevalent at any given point in time. This could help overtasked police forces engage with the criminal element. Unfortunately, some seasonal crimes, such as rape, vary so little and are so rarely reported that even targeted policing in this manner is unlikely to help. Reported rapes are extremely low, and the reduction in rape rates is very noisy though generally decreasing. Rape does seem to exhibit seasonality, as is shown in Fig 4 but the size of the oscillation is too small to make a difference in policing strategy and is very noisy from the small amount of data. Uncovering seasonal variations in crime can help us to understand the expected crime rates at a given point in time, and aid in deploying police resources.

While exogenous factors in the form of external forcing are certainly important in pinning these oscillations to a yearly cycle, a stochastic ecological model based on predator-prey relationships, specifically the Lotka–Volterra equations, can successfully reproduce the oscillations and noise in these cycles. The predator, in this case a criminal act, feeds off of prey, or criminal targets, thus raising the crime rate but reducing the number of possible targets. As the criminals generally only encounter a small number of targets, the effective number of prey in the system should be quite small. As an example, burglars generally will choose targets from past successful burglaries, and will limit their search to a fairly limited region in space [38, 39]. Thus of the many thousands of homes in a metropolitan area, only a limited number of them are viable targets. By applying our model to several crime data sets, we demonstrate that ecological factors alone can reproduce the variability in crime’s seasonality. However, we emphasize that external factors certainly play an important part in the seasons of crime, but their effect can be overwhelmed by stochasticity and human ecology.

Stochastic and social factors create dramatic variations in crime rates from year to year. These effects, coupled with low levels of daily crime, can overwhelm the dependence that hostile behavior has on temperature fluctuations. Though mild correlations can be found between spiking crime rates and aberrations in temperature, crime’s seasonality is not solely determined by the seasonal shifts in weather.

## Supporting information

S1 File Daily crime data for Los Angeles. Daily crime data for Los Angeles by crime type.
(CSV)
